# The Efficacy of Naïve versus Modified Mesenchymal Stem Cells in Improving Muscle Function in Duchenne Muscular Dystrophy: A Systematic Review

**DOI:** 10.3390/biomedicines9091097

**Published:** 2021-08-27

**Authors:** Oscar Yuan-Jie Shen, Yi-Fan Chen, Hong-Tao Xu, Chien-Wei Lee

**Affiliations:** 1 Faculty of Medicine, The Chinese University of Hong Kong, Hong Kong 999077, China; oscar.shen11@gmail.com; 2 The Ph.D. Program for Translational Medicine, College of Medical Science and Technology, Taipei Medical University, Taipei 11031, Taiwan; evan.yifan@tmu.edu.tw; 3 Master Program in Clinical Genomics and Proteomics, School of Pharmacy, Taipei Medical University, Taipei 11031, Taiwan; 4 Department of Orthopaedics and Traumatology, Faculty of Medicine, Prince of Wales Hospital, The Chinese University of Hong Kong, Hong Kong 999077, China; xhtsmed@gmail.com; 5 Institute for Tissue Engineering and Regenerative Medicine, The Chinese University of Hong Kong, Hong Kong 999077, China; 6 School of Biomedical Sciences, Faculty of Medicine, The Chinese University of Hong Kong, Hong Kong 999077, China

**Keywords:** mesenchymal stem cells Duchenne muscular dystrophy, muscle function, fibrosis, regenerative cell therapy, contraction-induced injury

## Abstract

As one of the most common genetic conditions, Duchenne muscular dystrophy (DMD) is a fatal disease caused by a recessive mutation resulting in muscle weakness in both voluntary and involuntary muscles and, eventually, in death because of cardiovascular failure. Currently, there is no pharmacologically curative treatment of DMD, but there is evidence supporting that mesenchymal stem cells (MSCs) are a novel solution for treating DMD. This systematic review focused on elucidating the therapeutic efficacy of MSCs on the DMD in vivo model. A key issue of previous studies was the material-choice, naïve MSCs or modified MSCs; modified MSCs are activated by culture methods or genetic modification. In summary, MSCs seem to improve pulmonary and cardiac functions and thereby improve survival regardless of them being naïve or modified. The improved function of distal skeletal muscles was observed only with primed MSCs treatment but not naïve MSCs. While MSCs can provide significant benefits to DMD mouse models, there is little to no data on the results in human patients. Due to the limited number of human studies, the differences in study design, and the insufficient understanding of mechanisms of action, more rigorous comparative trials are needed to elucidate which types of MSCs and modifications have optimal therapeutic potential.

## 1. Introduction

DMD is an X-linked recessive pediatric disorder and is one of the most frequent genetic conditions, affecting approximately 1 in 3500 male births worldwide [1]. The dystrophin mutation at locus Xp21 results in a sarcolemma lacking dystrophin, which is a vital structural link between the extracellular matrix and the cytoskeletal proteins [2]. The absence of dystrophin destabilizes the sarcolemma-cytoskeleton architecture, causing myofibers to be highly susceptible to contraction-associated mechanical stress. Subsequent myocyte death results in the inflammation and fibrosis of muscle [2].

Patients with Duchenne’s are diagnosed at the average age of 5, and more than 90% are in wheelchairs by age 15 [3]. Dystrophin is demonstrated to be involved in brain development, and patients with DMD are more likely to have conditions such as attention deficit hyperactivity disorder, autism, and seizure disorders [4]. Although the life is extended by supportive interventions, a substantial economic burden remains, not only for DMD patients but also the whole healthcare system, with the US household burden in 2012 estimated to be $58,440 to $71,900 and a conservative societal burden estimated between $80,120 and $120,910 per year per patient, which does not include mortality—likely a major cost component because of the low life expectancy associated with DMD [5]. Due to improvements in supportive interventions, patients can now survive into their 40s, generally succumbing to respiratory or cardiac failure [6]. Skeletal muscle structure and function are abnormal in individuals with heart failure, which contributes to deconditioning and exercise intolerance. Heart failure patients also exhibit attenuated peripheral vascular responses to exercise and reduced respiratory muscle endurance. Respiratory failure appears to occur secondary to heart failure rather than directly due to skeletal muscle weakness. Most strategies, however, such as corticosteroids, although slightly improve prognosis, have numerous adverse effects and are by no means curative for DMD [7].

Since there is no definitive cure for DMD, current therapeutics are palliative and focus on managing symptoms and slowing disease progression [8]. Corticosteroids are the most used medication for DMD, as they reduce inflammation in the muscles, help the heart and lungs remain stronger for longer, and reduce the chance of severe scoliosis [9]. Unfortunately, they are also associated with numerous adverse effects, including weight gain, osteopenia, growth impairment, and behavioral disturbances [10]. Myostatin inhibition is currently being evaluated in clinical trials for treating DMD, as myostatin normally limits muscle growth. Inhibiting myostatin would therefore increase muscle mass in patients and may delay the effects of muscle degeneration, but some preliminary studies have shown that direct treatment with myostatin inhibitors failed to improve outcomes [11]. As muscle fibers are damaged in DMD, fibrosis occurs and replaces muscle with fat and connective tissue, preventing muscles from working normally [12]. Clinical trials are being conducted to determine whether medications targeting the fibrosis process can preserve muscle integrity. There are also some therapies being investigated for specific mutations causing DMD, including exon-skipping and nonsense mutation suppression that increase the expression of dystrophin [13,14,15].

Stem cells are an attractive therapy for DMD because they could theoretically (1) replace damaged myofibers, (2) increase the expression of dystrophin, and (3) modulate the inflammatory effects, which directly results in fibrosis and muscular dysfunction [16]. MSC properties, including immunomodulation, non-tumorgenicity, no ethic issues, enhanced regeneration, in vitro expansion ability, and even anti-senescence ability, make them promising in the treatment of DMD [17]. In this study, we focus on the clinical significance of MSC treatment; therefore, direct muscle function measurements were emphasized rather than secondary outcomes such as protein expression, muscle fibrosis, and creatine kinase levels. A systemic understanding of the use of MSCs in DMD would provide insights into new treatment methods that can be investigated and elucidate the benefits of the methods that have already been tested. With advances in stem cell therapy, patients with DMD will hopefully have even longer lifespans and there will be a better quality of life for patients and caregivers.

## 2. Materials and Methods

The Preferred Reporting Items for Systematic Reviews and Meta-Analyses (PRISMA) statement was used for this article. Meanwhile, the Cochrane handbook was selected as the guidelines for the study protocol.

### 2.1. Search Strategy and Study Selection

We searched PubMed and Web of Science to select studies. We searched for MeSH terms and other related turning relating to “Mesenchymal Stem Cells”—which includes mesenchymal stromal cells and mesenchymal progenitors—and “Duchenne Muscular Dystrophy” in the title and abstract field. The details of selected search terms and search procedures are available in the Supplementary Material (Supplementary File). Additional studies were also located by searching papers referenced in listed articles. Studies identified through the search were combined, and any duplicates were removed. Then, titles and abstracts were screened before the in-detail review of full-text articles. Of the remaining studies, we examined their full full-text articles for inclusion in the study.

**Figure 1:**
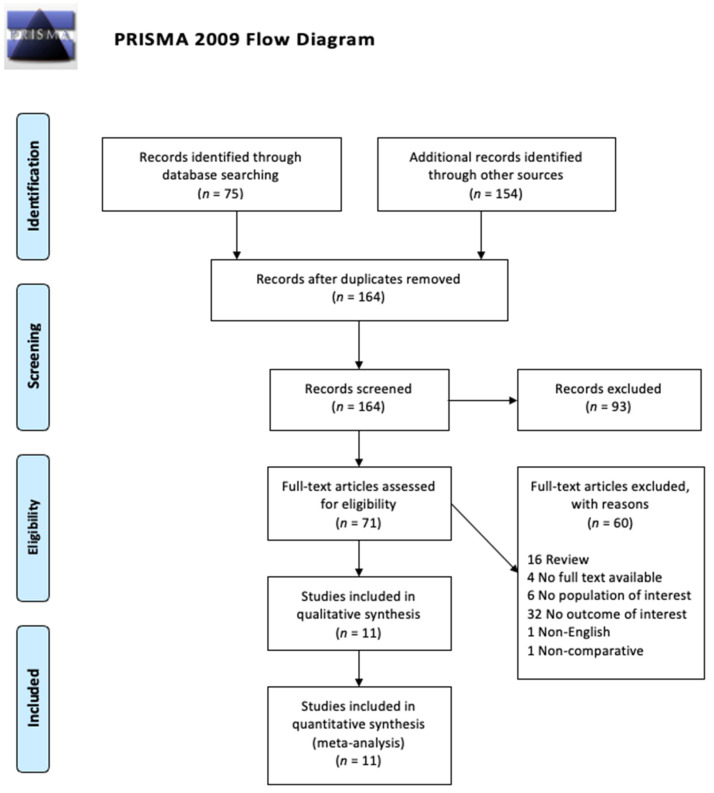
PRISMA Flow Diagram for the study selection process.

The search resulted in 258 studies across both data bases with a total of 66 duplicates. Our inclusion criteria were: full-text available, written in English, published in the last 20 years, contained original data, and related to MSCs and DMD. Our exclusion criteria were: text not in English, full-text not available, no direct measurement of muscle function, case reports, case series, reviews, articles, letters, and chapters. The criteria are illustrated in Table 1. All articles were screened by reading their titles and abstracts first. A total of 109 studies were removed because they did not fulfill the inclusion criteria or they met the exclusion criteria. The 53 remaining studies had their full text reviewed, and 44 of them were excluded after reviewing for various reasons—the most common being not having muscle function as a measured outcome. The 11 remaining studies underwent secondary full-text review and were confirmed as fitting the inclusion criteria. The flow chart of the selected studies selection process—based on the PRISMA 2009 Flow Diagram—is shown in Figure 1.

### 2.2. Data Extraction

All relevant data were extracted including: author information, published journal, year of publication, cell source, subject type, cell harvesting and processing method, injection method, and measurement methodology. Measurement methodology included tests to assess muscle function, PCRs, immunohistochemistry, western blots, and histological analysis. The details of results extraction consisted of variable/control group descriptions, measured effects on muscle function, and conclusions.

### 2.3. Methodological Quality Assessment

Methodological assessment is an essential procedure to exclude articles that could potentially have a higher degree of bias. The 11 identified studies were reviewed, and all included data were analyzed based on study heterogeneity and methodological quality. Due to the heterogeneity of the nature and measurement across studies, a meta-analysis could not be performed. The selected studies were assessed with the quality scoring system proposed by Slim et al. called the Methodological Item for Non-Randomized Studies (MINORS) tool [18]. The MINORS tool gives each article 0–2 points on up to 12 aspects, with a maximum score of 16 for non-comparative studies and a maximum score of 24 for comparative studies. Detailed scoring can be found in the Supplementary Material (Supplementary File).

## 3. Results

### 3.1. Study of Methodology Quality Assessment

According to the MINORS tool, all of the studies included had an extremely high score, with all comparative studies receiving a score of 22 out of a possible 24 and the only non-comparative study receiving a score of 13 out of 16. The scores are listed in Table 2. The high scores are likely since all the comparative studies used mice, which have established progenies that mimic Duchenne muscular dystrophy. Due to the well-controlled nature of all the studies, their scores were all remarkably high. None of the studies scored points regarding the prospective calculation of the study size needed for statistical significance. Using information on the size of the detectable difference of interest with a calculation of 95% confidence interval, according to the expected incidence of the outcome event, and information about the level for statistical significance and estimates of power when comparing the outcomes, an appropriate study size can be estimated to achieve statistical significance in outcomes.

### 3.2. Study of Characteristics

Detailed characteristics of selected studies can be found in Table 3. Of the selected 11 studies, one was published in 2009, one in 2010, one in 2011, one in 2014, one in 2015, two in 2018, one in 2019, and two in 2021. Only one was non-comparative and was also the only study done on human patients with Duchenne Muscular Dystrophy (Dai et al. [19]). Nine of the studies experimented on x-linked muscular dystrophy (mdx) mice, which are considered equivalent to Duchenne muscular dystrophy, with six using MSCs from humans (Siemionow M et al. (2019) [20], Pang et al. [21], Valadares et al. [22], Esper et al. [23], Siemionow M et al. (2021) [24], and Nitahara-Kashara et al. [25]), three using cells from Sprague-Dawley rats (Geng et al. [26], Li et al. [27], and Nitahara-Kashara et al. [25]), and three studies using cells from utrophin/dystrophin double knockout (dko) mice. Mdx are the most widely used animal model for DMD research overall. Their lifespans are relatively normal compared to those of individuals with DMD. Skeletal muscle changes only begin occurring between 3–6 weeks, with severe dystrophic phenotypes such as muscle wasting, scoliosis, and heart failure only occurring at 15 months or older [30]. Dko mice are distinct from mdx mice as mdx still have functional dystrophin. Mdx mice are hypothesized to regenerate skeletal muscle to some extent by replacing dystrophin with utrophin, resulting in a much milder disease state with lifespans comparable to those of wild-type mice. Dko mice have a much more severe form of the disease that mimics the progression of DMD and may be considered a superior model for DMD [31].

One study used MSCs from healthy beagle dogs. Six studies involved modified MSCs. MSCs were considered modified if they were processed or modified in any way beyond purely isolating or inducing differentiation in the cells, including adjuvant therapies. Siemionov et al. (2019) [20] created fused cells using a combination of MSCs from mdx mice and myoblasts from wild type and mdx mice. Ruehle et al. [28] used single-cell MSCs and aggregated MSCs using 3D culture. Geng et al. induced the differentiation of bone marrow MSCs and primed them with or without myostatin antibodies. Esper et al. used false aquapuncture with MSCs and true aquapuncture with MSCs. Siemionov et al. (2021) [24] also used fused cells using human MSCs and myoblasts. Nitahara-Kashara et al. used adenoviruses to transduce IL-10 vectors into some MSCs. Two studies investigated cardiac function using echocardiography and/or electromyography (Dai et al. [19], Siemionov et al. (2019) [20]), and one study investigated respiratory function using spirometry (Dai et al.). Four studies investigated muscle function using the rotarod performance test as a method (Pang et al. [21], Valadares et al. [22], Geng et al. [26], Li et al. [27]), three studies used electrodes to measure muscle function (Siemionov et al. (2021) [24], Ruehle et al. [28], Rousseau et al. [29]), and two studies used a wire test to measure motor function (Esper et al. [23], Siemionov et al. (2021) [24]). All of the studies also measured other surrogates in conjunction with immunohistochemical and/or histological analysis to confirm muscle function.

### 3.3. Cell Management and Injection

Siemionow et al. (2019) [20] harvested myoblasts from the hind limb muscles of both wild type and mdx mice. MSCs were harvested from the femur and tibia of mdx mice only. The fusion cells MBwt/MBmdx and MBwt/MSCmdx were created. Then, 0.25 × 10^6^ fused cells were given via 60 μL systemic-intraosseous saline injections into the femoral bone of mdx mice. Dai et al. [19] obtained Wharton-Jelly MSCs from consenting patients delivering full-term infants by Caesarian sections and injected 2 × 10^6^ cells/kg/dose every 2 weeks for 4 months, alternating between systemic intra-arterial administration and local intramuscular injections in patients with DMD. Ruehle et al. [28] used MSCs from C57BL/6 mice and formed MSCs into aggregates by centrifuging and incubating overnight to form spheroidal aggregates. The mice received 100 μL injections containing MSC aggregates, MSC single cells, or saline. Furthermore, 5 × 10^5^ MSCs were in the injection each mouse received except for the saline group. Geng et al. [26] isolated bone marrow MSCs from the femur and tibia of Sprague-Dawley rats and induced myogenic differentiation with or without 10–100 μg/mL polyclonal anti-myostatin antibody. Then, 1.2 × 10^7^ MSCs of the mixture were infused per mouse through the tail vein. One day before transplantation, mdx mice in the Ab transplantation group were injected intraperitoneally with anti-myostatin antibodies (6 mg/kg/week). Li et al. [27] extracted bone marrow MSCs from the femurs and tibias of male Sprague-Dawley rats. The cell density was adjusted to 1 × 10^7^ cells/mL. A volume of 0.5 mL MSCs suspended in PBS was administered via tail vein injection into each experimental utrophin/dystrophin-deficient double knockout mouse, and matched controls were administered equivalent injections of PBS. Pang et al. [21] induced the myogenic differentiation of embryonic-like stem cells (ELSCs) and MSCs by culturing them in myogenic differentiation medium (Lonza Group, Basel, Switzerland) for 10 days. Then, 2 × 10^6^ ELSCs or MSCs in 500 μL of saline were injected into each mouse through the tail vein. For controls, 500 μL of saline was injected through the tail vein. Valadares et al. [22] isolated fibroblasts, myoblasts, endometrial pericytes, fallopian tube pericytes, adipose pericytes, and muscle pericytes from a single 46-year-old healthy female donor. Then, 1 × 10^6^ cells (or vehicle) were injected intraperitoneally into the mice once a week for 8 weeks without any immunosuppression. Esper et al. [23] isolated MSCs from human deciduous for pulp digestion. After trypsinization, cells were washed twice and 1 × 10^4^ cells were suspended in 20 μL of saline and injected once every three weeks. Rousseau et al. [29] dissected muscles from the arms and legs of wild type and mdx mice and digested with collagenase and dispase to isolate muscle progenitor cells. The left and right extensor digitorum longus were injected in several sites with a total of 1.5 × 10^6^ cells injected per muscle. Siemionow et al. (2021) [24] used fused human myoblasts and MSCs (MB^N^/MSC^N^) and injected a total of 0.25 × 10^6^ of these fused cells into the left gastrocnemius of the mice with six injections. Nitahara-Kashara et al. [25] isolated MSCs from Sprague-Dawley rat bone marrow and transduced them with a luciferase-expressing retroviral vector, creating luciferase-expressing MSCs (Luc-MSCs). Canine CD271 + MSCs were transduced with a luciferase-expressing retroviral followed by transduction with enhanced green fluorescent proteins (eGFPs) or MyoD-expressing adenoviral vectors. All MSCs and human dental pulp stem cells (hDPSCs) were transduced with AAV1/eGFP or control AAV1/IL-10 vectors. Then, 5.0–10.0 × 10^6^ luciferase-expressing rat MSCs were injected intramuscularly into the right or left hindlimb muscle of NOD/SCID mice pretreated with cardiotoxin 1 day before treatment. Then, 1.0 × 10^7^ of both eGFP-MSCs and IL-10-MSCs were injected into the right and left hindlimb, respectively. Five days before treatment, healthy beagle dogs had cardiotoxin injected into the tibialis anterior. A total of 2.4–2.7 × 10^7^ cells/2 mL of AAV1/IL-10-transduced Luc-CD271 + MSCs were injected into their muscles on days 0 and 50 without immunosuppression. hDPSCs or AAV1/IL-10-transduced hDPSCs (4.0 × 10^6^ cells/mL/kg body weight at a rate of 1 mL/min) were administered intravenously into the canine muscular dystrophy model (CXMD_J_).

### 3.4. Measurement Instruments

For classifying results, the studies used a variety of measurement instruments, including histological analysis, immunohistochemistry, echocardiography, electromyography, spirometry, fluorescent in situ hybridization, servomotor, electrodes, cell immunomodulatory factor quantification, RT-PCR, rotarod tests, nicotinamide adenine dinucleotide phosphate fluorescence kit followed by spectrophotometry to detect creatine kinase, Western blot, and PCR. The detailed measurement instruments used in each study are listed in Table 3.

### 3.5. Experimental Variables and Controls

In Siemionow et al.’s (2019) study [20], the experimental variables were fused cells created with myoblasts from wild type and mdx mice (MB^wt^/MB^mdx^), fused cells created with myoblasts from wild type mice and MSCs from mdx mice (MSC^mdx^), and the simultaneous injection of wild type myoblasts and mdx myoblasts (MB^wt^ + MB^mdx^) and wild type myoblasts and mdx MSCs (MB^wt^ + MSC^mdx^) into mdx mice. Wild type mice with no treatment and mdx mice with saline injection served as controls. In Dai et al.’s [19] study, they did not perform any controls and used human Wharton jelly-derived MSCs to treat human patients with DMD. Ruehle et al. [28] used mouse-derived single-cell MSCs and aggregated MSCs to treat mdx mice without myotoxic injury and wild type mice with myotoxic injury and compared it to both types of mice receiving saline injections. Geng et al. [26] compared rat bone marrow MSC transplant +/− myostatin antibody into mdx mice to mdx mice that received no transplant. Li et al. compared rat MSC transplantation into double knockout (dko) mice to wild type mice with PBS injections and dko mice with only PBS injections (no MSCs). Pang et al. [21] performed embryonic stem cell and MSC injection into dko with saline injection only in dko mice as a control. Valadares et al. [22] injected a variety of cell types (fibroblast, myoblast, endometrial pericyte, fallopian tube pericyte, adipose pericyte, and adipose pericyte) into dko mice and used vehicle (HBSS) injection only in dko mice as a control. Esper et al. [23] were interested in the therapeutic effects of aquapuncture and used false aquapuncture with stem cells from human exfoliated deciduous teeth (SHED), true aquapuncture with saline, and true aquapuncture with SHED in mice with no aquapuncture as a control. Rousseau et al. [29] transplanted mdx mice with muscle progenitor cells derived from normal 10 J mice and mdx mice. They compared them to mdx mice with no treatment and mdx mice injected with cardiotoxin. Siemionow et al. (2021) [24] injected fused MB^N^/MSC^N^ cells and unfused MB^N^ + MSC^N^ cells into mdx mice, comparing them with each other and vehicle injections as the control. Nitahara-Kashara et al. [25] compared mice transplanted with IL-10 MSCs with those transplanted with eGFP-MSCs, healthy beagles transplanted with cardiotoxin and IL-10-Luc-CD271 + MSCs with healthy beagles transplanted with cardiotoxin and MyoD-Luc-CD271 + MSCs, and CXMD_J_ transplanted with IL-10-hDPSCs with those transplanted with normal hDPSCs. The details are summarized in Table 4.

### 3.6. Laboratory Effects and Proposed Mechanisms

Studies involving modified MSCs are marked with an asterisk (*).

#### 3.6.1. MSCs Improved Life Expectancy and Limb Function

All studies were able to find some benefit in MSCs regardless of their methodology. Valadares et al.’s [22] results showed the least benefits from MSCs, as the only benefit found was an increased lifespan in mice treated with adipose-derived pericytes, and there is no factor that explains this finding. Three studies (Dai et al. [19], Valadares et al. [22], Li et al. [27]) measured both life expectancy—or its substitutes (cardiac and/or pulmonary function)—and limb function. Dai et al. [19] found that MSCs improved functional vital capacity (FVC) and forced expiration volume in one second (FEV1) on spirometry and improved equal ejection fraction on echocardiography, but there was no significant difference in muscle strength on electromyography. Li et al. [27] were able to increase lifespan by over 50%, although still less than half of the lifespan of normal control mice. Locomotor function was greatly improved. Valadares et al. [22] found increased lifespan in mice treated with adipose-derived pericytes and found no improved muscle function in any other groups. Siemionow et al.’s (2019)* [20] study was the only one to measure cardiac function using echocardiography, and it found significant improvements in ejection fraction and fractional shortening. The remaining seven studies (Pang et al. [21], Esper et al.* [23], Siemionow et al. (2021)* [24], Nitahara-Kashara et al.* [25], Geng et al.* [26], Ruehle et al.* [28], Rousseau et al. [29]) only measured limb function, and all of them were able to find improvements.

Two studies (Dai et al. [19], Valadares et al. [22]) failed to show increased skeletal muscle function, but did find benefits to survival. Dai et al. [19] used human Wharton jelly-derived MSCs and found that there was no significant difference in muscle strength measured using a Powertrak HandHeld Dynamometer despite a significant increase in the amplitude measured via electromyography of the right and left soleus when injected with Wharton jelly-derived MSCs compared to controls. There was an increase in the dystrophin measured through immunohistochemistry. Valadares et al. [22] increased life expectancy with adipose-derived pericytes, but there was no improvement in muscle function in mdx mice.

#### 3.6.2. MSC Induced Myogenic Differentiation Is an Important Factor in Improving Outcomes

Four of the studies (Siemionow et al. (2019)* [20], Pang et al. [21], and Siemionow et al. (2021)* [24], Geng et al.* [26]) showed that the ability of cells to differentiate was important in improving muscle function. Siemionow et al. (2019)* [20] demonstrated this, as fused myoblasts from mdx and wild type mice had increased ejection fractions and fractional shortening on the echocardiogram compared to mice injected with the myoblasts of wild type mice and the MSCs of mdx mice. The immunofluorescence staining of dystrophin also found much higher levels in tissue treated with MB^wt^/MB^mdx^ fusion cells compared to MB^wt^/MSC^wt^. Geng et al.* [26] also demonstrated that differentiation was essential. Although MSCs induced toward myogenic differentiation failed to improve muscle function despite expressing myostatin on immunofluorescence and RT-PCR, MSCs that were subsequently treated with myostatin antibodies had greatly improved muscle function. This suggests that the benefits of MSCs are not directly from their differentiation but rather from their ability to induce differentiation in tissue, which benefits from the presence of myostatin antibodies. Through cell culture, Geng et al.* [26] showed that increased myostatin antibody concentration is associated with decreased MSC differentiation into adipocytes on cell culture and increased myogenesis with increased MyoD expression on RT-PCR. Pang et al. [21] found both ELSCs and MSCs were able to provide significant benefits. ELSCs outperformance of MSCs was attributed to the increased differentiation potential of ELSCs, as evidenced by more MHC and myogenin expression on cultures. Valadares et al. [22] experimented with various sources of pericytes and found that only adipose-derived pericytes increased the life expectancy of the DMD mice. They also analyzed transcript and protein expression and demonstrated the low myogenic potential of pericytes. In Siemionow et al. (2021)* [24], the skeletal myosin heavy chain expression in differentiated myoblasts was significantly higher than in undifferentiated myoblasts. The differentiation of MSCs down myogenic lineages is highly controversial, and although some of the included studies show myogenic-like differentiation, there is a possibility of it being an artifact due to the contamination of MSC cultures with myogenic cells.

#### 3.6.3. MSC Incorporation and Proliferation in Muscle Is Correlated with Improved Outcomes

Six studies (Siemionow et al. (2019)* [20], Valadares et al. [22], Siemionow et al. (2021)* [24], Nitahara-Kashara et al.* [25], Ruehle et al.* [28], Rousseau et al. [29]) showed that stem cell incorporation and proliferation in muscle tissue was essential. The fused cells used by Siemionow et al. (2019) [20] demonstrated significant improvement in cardiac function. Echocardiography and histological analysis showed less fibrosis than saline or treatment with non-fused cells. These findings were attributed to fused cells having better engraftment potential and lower levels of rejection. Ruehle et al. [28] found that single-cell MSCs failed to improve the isometric torque of the ankle plantar flexor muscles via servomotor and could not be reliably identified on histology at any point during the experiment. Aggregated MSCs were able to improve muscle function and could be detected on histology for up to 7 days in cardiotoxin-injured wild type muscle and up to 3 days in mdx muscle. Aggregated MSCs also had higher levels of growth factors (IL-6, PGE-2, and HGF) on ELISAs, which would facilitate proliferation. Valadares et al. [22] failed to find any transplanted human cells in the mice gastrocnemius on histology and also failed to find any improvements in function as well. Rousseau et al. [29] found mdx mice transplanted with 10 J mesenchymal progenitor cells (MPCs) had increased protection against eccentric contractions. That group was the only one in the study that had increased dystrophin positive fibers on immunohistochemistry due to the fusion of donor MPCs and host muscle fibers. Siemionow et al. (2021) [24] demonstrated that fusing myoblasts with MSCs significantly increased both dystrophin and skeletal myosin heavy chain expression in vivo compared to injecting unfused cells. The increase is likely due to a synergistic effect of the increased differentiation of the myoblasts and the higher proliferation potential of the MSCs. Nitahara-Kashara et al.* [25] found that IL-10 expressing MSCs had significantly more cell survival and were more effective at enhancing post-transplantation retention compared to controls in their mice models. While muscle function was not measured in mice, the canine models they used also demonstrated higher retention of IL-10 MSCs in muscles with improvements in motor function.

#### 3.6.4. Improved Exercise Resistance as a Major Mechanism of MSC’s Benefits

Nitahara-Kashara et al.* [25] noted that while they found that both hDPSC and IL-10-hDPSC-treated CXMD_J_ exhibited no significant difference in running speed, dogs treated with IL-10-hDPSC exhibited significantly higher torque values, lower CK & lactic acid levels post-exercise, and higher muscle mass than untreated and normal hDPSC groups. As the CXMD_J_ were sacrificed after 8 weeks of treatment, further investigation is necessary to determine if IL-10-hDPSC would maintain running speed longer than regular hDPSC due to the evidence of improved exercise resistance.

On a related note, Rousseau et al. [29] found no significant force difference between any of the groups they tested. Mdx mice with 10 J MPCs transplanted were found to have increased muscle protection against eccentric contractions through their eccentric contraction protocol, but no improvements in the performance of their isometric contraction protocol were observed. Immunohistochemistry was able to detect significantly higher levels of dystrophin in mdx mice transplanted with 10 J MPCs compared to any other groups, suggesting that replacing dystrophin increases resistance to exercise and contraction-induced injury but not the strength of the muscle. They unfortunately did not measure cardiac function, which if improved would provide strong evidence for dystrophin being protective against contraction-induced injury, as the heart is most likely to sustain eccentric damage during normal activity.

## 4. Discussion

Despite DMD being first described in the 1860s, little was known about the pathophysiology of the disease until the late 1980s, and even now, corticosteroids are the most common method of treatment that provides symptomatic control and attempts to slow progression without directly addressing the root cause of DMD—a lack of dystrophin [32]. Finding an effective treatment to improve quality of life and life expectancy would provide immense benefits to patients and society at large. MSC-based therapies provide attractive therapeutic options due to their relative abundance and ability to mitigate inflammation—the main driver of fibrosis and loss of muscle function in patients—directly and through its immunomodulatory effects [33]. Through this study, we consolidated all studies evaluating the effectiveness of MSCs in treating DMD. We found that modifying MSCs to promote differentiation and proliferation are likely to improve the effectiveness of MSC treatment. Our study also highlights the necessity for standardized protocols and further studies on which modifications are most effective to maximize the therapeutic potential of MSCs.

We found that the most common issue with transplanted naïve MSCs was that they have short-term survival in vivo, as evidenced by several studies (Valadres et al. [22], Nitahara-Kashara et al. [25], Ruehle et al. [28]) failing to find immigrated MSCs in host tissue. Furthermore, many of the studies only investigate local injections of MSCs (Esper et al. [23], Siemionow et al. (2021) [24], Nitahara-Kashara et al. [25], Ruehle et al. [28], Rousseau et al. [29]). However, due to the systemic nature of DMD, a systemic delivery method is needed, and thus the optimal method of ensuring MSC incorporation is unclear. Based on our study, modifying MSCs to promote differentiation (i.e., fused cells), aggregation (i.e., aggregated MSCs), or proliferation (i.e., myostatin antibodies) tended to yield better outcomes and was more likely to result in direct improvements in muscle functions and not just in the markers of muscle function such as creatine kinase. Also of note is that none of the included studies worked with skeletal muscle resident MSCs. Bone marrow MSCs were predominantly used, which could be a potential source of weak results in some studies.

The exact mechanism of MSCs improving muscle function is not clear either. MSCs could differentiate myogenically or augment myogenic cell’s abilities to regenerate. The selected studies had some evidence of both hypotheses, and future studies will hopefully establish the pathways involved that would have significant implications for MSC modification.

Two studies (Siemionow et al. (2019) [20], Valadares et al. [22]) failed to show benefits in skeletal muscle function but found a longer life expectancy in mdx mice treated with MSCs. While those studies did not directly address the reason for the longer life expectancy, MSCs have been shown to reduce cellular senescence in premature mice. Rousseau et al. found that MSCs improved resistance to contraction-induced injury, which would theoretically improve life expectancy but was unfortunately not measured.

This review was limited by the wide range of protocols used by the included studies. MSCs were harvested, processed, and delivered differently in each study. For example, some delivered MSCs systemically, others with local injections, and one even administered MSCs intraperitoneally. Modified MSCs were only modified in one manner, and there was essentially no overlap and comparison in the modifications between studies. These differences make direct comparisons between the studies challenging and prevents us from studying the relative effectiveness of different modifications. Therefore, we are unable to make solid and generalizable claims about the efficacy of different methods. Most of these experiments were performed in animal models, and human trials are still very rare, as evidenced by only one human study in our analysis. Whether these findings are transferable to humans requires further investigation.

Future studies should focus on direct comparisons of various modifications or different combinations of them to optimize the use of MSCs. Creating a standardized protocol for the harvesting, processing, and delivery of MSCs would also be helpful to allow for more direct comparisons between studies. Furthermore, comparing different cell sources and cell numbers to maximize MSC effects in treatment is necessary as well. More human trials should be attempted to ensure that results from animal models are transferable to clinical practice. According to ClinicalTrials.gov, there are only three clinical trials investigating the use of MSCs in DMD, with no results released. This demonstrates the urgent need for new developments in therapeutics.

Our study establishes that MSCs have therapeutic potential in DMD. Modified MSCs apparently provide more benefits than naïve MSCs, although which modifications are optimal have yet to be confirmed. We have identified three characteristics of modified MSCs that have been associated with better outcomes, namely, increased myogenic differentiation, aggregation in the target tissue, and proliferation potential. Using highly optimized MSCs to treat DMD could revolutionize treatment—which is largely palliative—for the most common cause of muscular dystrophy and could provide the groundwork for treating other muscular dystrophies using MSCs.

## Figures and Tables

**Table 1:** Inclusion and exclusion criteria for studies.

Inclusion Criteria	Exclusion Criteria
Full-text available	Full text not available
Written in English	Text not in English
Articles published in the last 20 years	No direct measurement of muscle function
Articles containing original data	Case reports, case series, reviews, letters, chapters
Studies related to mesenchymal stem cells and Duchenne muscular dystrophy	

**Table 2:** MINORS score of selected studies.

Study	MINORS Score
Dai A. et al. (2018) [19]	13 of 16
Siemionow M. et al. (2019) [20]	22 of 24
Pang R. et al. (2014) [21]	22 of 24
Valadares M. et al. (2014) [22]	22 of 24
Esper G. et al. (2015) [23]	22 of 24
Siemionow M. et al. (2021) [24]	22 of 24
Nitahara-Kashara et al. (2021) [25]	22 of 24
Geng J et al. (2009) [26]	22 of 24
Li Z. et al. (2011) [27]	22 of 24
Ruehle M. et al. (2018) [28]	22 of 24
Rousseau J. et al. (2010) [29]	22 of 24

**Table 3:** Characteristics of selected studies.

Author	Journal	Year	Compartive/Non-Comparative	Cell Source	Subject Type; aGe at Treatment	Modified/Unmodified	Cell Harvesting and Processing	Injection Method	Number of Independent Measurements	Measurement Methodology *	Reference
Dai A. et al. (2018) [19]	Degenerative Neurological and Neuromuscular Disease	2018	Non-comparative	Human	Human; 7 to 14 years old	Unmodified	Umbilical cords were obtained from consenting patients delivering full-term infants by Caesarian section	2 × 10^6^ cells/kg/dose injections every 2 weeks for 4 months, alternating between systemic intra-arterial administration and local intramuscular injections	5	**Electromyography; Spirometry; Echocardiography**; Immunohistochemical analysis; Fluorescent in situ hybridization	[19]
Siemionow M. et al. (2019) [20]	Stem Cell Reviews and Reports	2019	Comparative	mdx mice; snj (wild type) mice	Mdx mice; 8 weeks old	Modified	Myoblasts were harvested from the hind limb muscles of both wild type and mdx mice. MSCs were harvested from the femur and tibia of mdx mice only. The fusion cells MBwt/MBmdx and MBwt/MSCmdx were created. Some MBwt, MBmdx, and MSCmdx were saved	60 μL systemic-intraosseous saline injections to femoral bone	3	Histological and immunofluorescence of cardiac muscle cross sections; **Echocardiography Assessment**	[20]
Pang R. et al. (2014) [21]	Cytotherapy	2014	Comparative	Human	Mdx mice; 6 weeks old	Unmodified	To induce myogenic differentiation, cells were cultured in myogenic differentiation medium (Lonza, Basel, Switzerland) for 10 days.	Two million ELSCs or MSCs in 500 μL of saline were injected into each mouse through the tail vein. For control, 500 μL of saline was injected through the tail vein.	9	**Analysis of motor function via traction, rotating rod, and running wheel tests**; CK activity via NADPH fluorescence kit followed by spectrophotometry; Immunohistochemistry; Western blotting; RT-PCR; Histological analysis	[21]
Valadares M. et al. (2014) [22]	Stem Cell Reviews and Reports	2014	Comparative	Human	Mdx mice; 7 weeks old	Unmodified	Four tissue specimens from a 46-year-old healthy female donor, namely: adipose tissue, muscle, fallopian tubes and endometrium were obtained from total hysterectomy procedures. All pericyte populations were confirmed to have at least 75 % purity after sorting.	Injected intraperitonially with 1 million viable cells (or vehicle), once a week, for a total of 8 weeks, without any immunosuppression. These seven groups were comprised by animals receiving either: vehicle, fibroblasts, myoblasts, pericytes (endometrium), pericytes (fallopian tubes), pericytes (adipose tissue) and pericytes (muscle).	9	**Analysis of motor function via ambulation test, grip test, and rotarod**; Myogenic differentiation; qPCR; PCR; Western blot; Immunohistochemistry; Histological analysis	[22]
Esper G. et al. (2015) [23]	Evidence-Based Complementary and Alternative Medicine	2015	Comparative	Human	Mdx mice; 4 to 6 weeks old	Modified	MSCs derived from deciduous teeth were obtained using a modified Miura’s protocol with collagenase type I for pulp digestion	1 × 10^4^ cells suspended in 20 microliters saline injected every three weeks, totaling three injections via acupoint at Bladder 47, 49, and 52.	3	**Analysis of motor function via wire test**; CK-NAC; Histological analysis	[23]
Siemionow M. et al. (2021) [24]	Stem Cells and Development	2021	Comparative	Human	Mdx mice; 6 to 8 weeks old	Modified	Allogenic human myoblasts and normal human bone marrow-derived MSCs underwent fusion exvivo using polyethylene. Cells presenting double PKH26/PKH67 staining were sorted through fluoresence-activated cell sorting and used for in vitro and invivo analysis	0.25 × 10^6^ fused cells or 0.5 × 10^6^ of unfused cells were suspended in 60 microliters of Dulbecco’s phosphate-buffered saline. This volume was delivered into the left gastrocnemius of each mouse through six injections	6	**Analysis of motor function via wire hanging and grip strength test, in situ muscle force test, and ex vivo muscle force test**; Histological analysis; Immunofluoresence analysis	[24]
Nitahara-Kashara et al. (2021) [25]	Stem Cell Research & Therapy	2021	Comparative	Sprague-Dawley rats, Human, Beagle dogs	NOD/SCID mice, Beagle dogs, Canine X-linked muscular dystrophy model (CXMDj); 3 to 52 months old	Modified	MSCs isolated from Sprague-Dalwey rat bone marrow were transduced with a luciferase-expressing retroviral vector. Canine CD271 + MSCs were also transduced with a luciferase-expressing retroviral vector as well as an enhnaced green fluorescent protein (eGFP) or MyoD-expressing adenoviral vector. MSCs or human dental pulp stem cells (hDPSCs) were transduced with adeno-associated virus (AAV)/eGFP or control AAV1/IL-10 vectors	5.0–10.0 × 10^6^ luciferase-expressing rat MSCs were injected intramuscularly into the right or lift hindlimb muscle of NOD/SCID mice pretreated with cardiotoxin 1 day before treatment. 1.0 × 10^7^ of both eGFP-MSCs and IL-10-MSCs were injected into the right and left hindlimb, respectively. AAV1/IL-10 transduced Luc-CD271 + MSCs (2.4–2.7 × 10^7^ cells/2 mL) were injected into the muscles of healthy beagles on days 0 and 50 without immunosupressants. The tibialis anterior muscles were pretreated by injecting 10 nmol/kg of cardiotoxin. hDPSCs or AAV1/IL-10 transduced hDPSCs (4.0 × 10^6^ cells/mL/kg body weight at a rate of 1 mL/min) was adminstered intravenously into CXMDj using nine injections at two week intervals.	13	**Analysis of motor function via 15-m running time and counts of spontaneous locomotor activity**; Creatinine kinase; Alanine aminotransferase; Aspartate aminotransferase; blood urea nitrogen; Histological analysis; Immunohistochemical analysis; ELISA; Luciferase reporter assays; Biodistribution of MSCs; Cytokine/cytokine array; MRI	[25]
Geng J et al. (2009) [26]	Cytotherapy	2009	Comparative	Sprague-Dawley rats	Mdx mice; 7 to 9 weeks old	Modified	Femur and tibia bone marrow mesenchymal stem cells were incubated in complete medium containing 10 μM 5-AzaC for 24 h to induce differentiation, and incubated with or without 10–100 μg/mL polyclonal anti-myostatin Ab in differentiation medium	1.2 × 10^7^ MSC of the mixture were infused per mouse through the tail vein. One day before transplantation, mdx mice in the Ab transplantation group were injected intraperitoneally with anti-myostatin antibody (6 mg/kg/week).	5	Immunofluorescence analysis; RT-PCR; **Motor function via Rota-rod**; CK-NAC; Western blot	[26]
Li Z. et al. (2011) [27]	Cytotherapy	2011	Comparative	Sprague-Dawley rats	Mdx mice; 6 weeks old	Unmodified	Bone marrow MSCs were extracted from the femurs and tibias of male rats.	Cell density was adjusted to 1 × 10^7^/mL. A volume of 0.5 mL MSC suspended in PBS was administered via tail vein injection into each experimental dko mouse, and matched controls were administered equivalent injections of PBS.	5	**Analysis of motor function via traction, rotating rod, and running wheel tests**; Histological analysis; Detection of grafted cells	[27]
Ruehle M. et al. (2018) [28]	Journal of Tissue Engineering and Regenerative Medicine	2018	Comparative	Wild type C57BL/6 mice	Mdx mice; 12 weeks old	Modified	Mesenchymal stem cells were lentivirally GFP-labelled to confirm multipotency and proliferative capacity. Some cells were formed into aggregates by centrifuging and incubating overnight to form spheroidal aggregates	Mice received local 100 μL injections containing MSC aggregates, MSC single cells, or saline. 5 × 10^5^ MSCs were in each injection except for saline	4	**Peak isometric torque via servomotor and Pt-Ir needle electrodes**; Histological analysis; Cell immunomodulatory factor quantification	[28]
Rousseau J. et al. (2010) [29]	Cell Transplantation	2010	Comparative	Normal C57BL/10 J and C57BL/10 J mdx/mdx mice	Mdx mice; 12 month old	Unmodified	Muscles dissected from arms and legs were cut into small fragments and digested with collagenase and dispase. After 48 hrs in culture, Muscle precursor cells were frozen in nitrogen until transplantation.	A total of 1.5 million cells were injected in several sites throughout each of the left and right extensor digitorum longus	2	**Analysis of motor function via electrode stimulation in organ bath**; Immunohistochemistry	[29]

* Measurement methodologies considered to directly measure muscle function are bolded.

**Table 4:** Experimental variables, results, and conclusions of selected studies.

Study	Variables	Controls	Modified/Unmodified	Effects on Muscle	Conclusions
Siemionow M. et al. (2019) [20]	MBwt + MBmdx	Wild type with no treatment	Modified	Increased ejection fraction and fractional shortening in MBwt/MBmdx and MBwt/MSCmdx compared to saline injected mdx controls	Fused cells are more efficacious in countering the effects of systolic dysfunction due to better engraftment potential and lower levels of rejection.
	MBwt + MSCmdx	Mdx mice with saline injection only		Increased ejection fraction and fractional shortening in MBwt/MBmdx and MBwt/MSCmdx compared to not-fused cells	MSCs are effective at maintaining cardiac function
	MBwt/MBmdx			MBwt/MBmdx and MBwt/MSCmdx mice had ejection values 10% and 20% higher, respectively, compared to vehicle injected controls	
	MBwt/MSCmdx				
Dai A. et al. (2018) [19]	Human wharton jelly-derived mesenchymal stem cells	None	Unmodified	Increased FVC and FEV1 for all patients	Positive effect for vital functions
				Increased or equal ejection fraction for 8 out of 9 patients	Questionable benefits in terms motility despite drop in CK
				No statistically significant difference between pre and post measure of muscle strength	
				Significant difference between pre and posttreatment EMG measurement of amplitude in right and left suralis	
Ruehle M. et al. (2018) [28]	Wild-type mice with myotoxic injury injected with single cell MSCs	Wild-type mice with myotoxic injury with saline only injections	Modified	No difference in peak isometric torque of the ankle plantar flexor muscles prior to treatment	Single cell MSCs are not effective at improving muscle function
	Wild-type mice with myotoxic injury injected with aggregated MSC	Mdx mice with no myotoxic injury with saline only injections		Aggregate treated mice had significantly greater torque than saline for both types of mice	Aggregated MSCs are effective at improving muscle function
	Mdx mice with no myotoxic injury injected with single MSCs			Aggregate MSCs had greater peak isometric torque compared to single cell for both types of mice	Aggregated MSCs may have avoided rapid clearance from tissue and remained within muscle longer than single cells, as aggregates could be identified for up to 7 days in the cardiotoxin-injured wild-type mice and for up to 3 days in the dystrophic tissue, whereas single cells could not be reliably identified by histology at any time point
	Mdx mice with no myotoxic injury injected with aggregated MSCs			Single cell MSCs had the same peak isometric torque as saline	Wild-type mice with myotoxic injury and mdx mice do not have a significant impact on the efficacy of stem cells
Geng J et al. (2009) [26]	Rat bone marrow mesenchymal stem cells (transplant)	No transplant	Modified	No difference between the no transplant and transplant group	MSCs alone are ineffective at improving muscle function
	Rat bone marrow mesenchymal stem cells + myostatin antibody (Ab transplant)			Ab transplant group greatly improved motor function compared to no transplant and transplant	Inhibition of myostatin improves the MSC ability to improve muscle function
Li Z. et al. (2011) [27]	MSC transplantation	PBS injection only wild type	Unmodified	Until 15 weeks after transplantation, no abnormalities were observed in either the diet or behavior of transplanted mice	MSCs provide significant improvements in motor function and lifespan, although it is still significantly worse than normal mice
		PBS injection only dko mice		The median lifespan of normal control mice was significantly higher than both other groups, but the median lifespan of the transplantation group (35 wks) was significantly higher than that of the control mice (22 wks)	
				All motor function tests showed significant improvement in experimental mice compared to control groups. Normal control mice performed significantly better than experimental mice, though	
Pang R. et al. (2014) [21]	ELSC injection	Saline injection only dko mice	Unmodified	ELSC transplanted mice had significantly improved motor function in all tests compared with dko mice transplanted with MSCs or saline	ELSCs were superior to MSCs by every metric, even though MSCs were already significantly better than saline only injections. The increased myogenic differentiation of ELSCs may be responsible for this discrepancy
	MSC injection				
Valadares M. et al. (2014) [22]	Fibroblast injection	Vehicle (HBSS) injection only in dko mice	Unmodified	Endometrial-derived pericytes showed significant effects related to the age of the onset of injections. The younger the dko mice started being treated, the better the survival. This observation was not seen in any other treated groups	All cells were ineffective at engrafting and providing significant benefits to muscle function
	Myoblast injection			Only adipose derived pericytes increased life expectancy in mdx mice	
	Endometrial pericyte injection			Despite improved survival in adipose pericyte injected mice, none of the physical tests revealed differences between the groups	
	Fallopian tube pericyte injection			No human cells were found in any analyzed tissues and no difference in the HE stained sections of the gastrocnemius muscle	
	Adipose pericyte injection			No in vitro myogenic potential in pericytes derived from any tissue source was found	
	Muscle pericyte injection				
Esper G. et al. (2015) [23]	False aquapuncture with SHEDs	No aquapuncture	Modified	Strength improvement in mice with SHED/true acupoints and only slight improvement with saline/true acupoints and SHED/false acupoints compared to controls	Acupuncture and MSCs each have a beneficial effect in muscle force that can complement each other
	True aquapuncture with saline			Although creatine kinase decreased in all treatments, only SHED/true acupoint was able to improve force	
	True aquapuncture with SHEDs				
Rousseau J. et al. (2010) [29]	Mdx mice transplanted with 10 J MPCs	No treatment mdx mice	Unmodified	There was no significant force difference observed between the different groups, even those injected with cardiotoxin	Replacing dystrophin can increase resistance to exercise and contraction-induced injury but not sufficiently enoigh to increase the strength of the muscle
	Mdx mice transplanted with mdx MPCs	Mdx mice injected with cardiotoxin only		Mdx mice with 10 J MPCs transplanted increased muscle protection against eccentric contractions	
Siemionow M. et al. (2021) [24]	MB/MSC fused cells	DPBS injection only	Modified	MB/MSC fused cells significantly improved in vivo muscle force and reduced fibrosis compared with vehicle-injected controls and non fused MB and MSC cells	Fused cells were more effective at improving muscle function than unfused cells
	Not-fused MB and MSC				
Nitahara-Kashara et al. (2021) [25]	Mice transplanted with IL-10 MSCs	Mice transplanted with GFP-MSCs	Modified	Muscle function was not measured specifically in mice, but IL-10 expressing MSCs had significantly more cell survival and were more effective at enhancing post-transplantation retention. Il-10-Luc-MSCs were retained for more than 67 days after transplantation	Higher retention in early stages exerts a significant effect on long-term engraftment
	Beagles transplanted with cardiotoxin and IL-10-Luc-CD271 + MSCs	Beagles transplanted with cardiotoxin and MyoD-Luc-CD271 + MSCs		Lucisferase activity, which correlates to the number of MSCs, tended to be higher in IL-10-Luc-CD271 + MSCs. IL-10 levels in IL-10-Luc-CD271+ MSC treated tibialis anterior muscles increased, while those in MYoD-Luc-CD271 +MSC-treated muscles did not.	IL-10 expressing CD271+ MSCs could survive long term and engraft after intramuscular regeneration
	CXMDj transplanted with AAV1/IL-10-transduced hDPSCs	CXMDj transplanted with hDPSCs		Significantly higher torque was found in IL-10-hDPSC-treated CXMDj than in control CXMDj, which had similar values to hDPSC-treated CXMDj. hDPSC and IL-10-hDPSC-treated CXMDj maintained a 15-m running speed and were active at 3 to 12 months of age. Only IL-10-hDPSC was effective at reducing CK levels and lactic acid before and after exercise	Only IL-10-hDPSCs exert a protective effect against dystrophic damage caused by exercise
				IL-10-hDPSCs were only detected in the TA muscles and not in other organs. Dystrophin expression was undetectable in the muscle tissues of hDPSC-treated CXMDj	
				Il-10-hDPSCs decreased inflammation and necrotic/edematous lesions and increased the weight and area of muscles. hDPSCs significantly limited the infiltration of nuclei, indicating a midler phenotype	

## Data Availability

Not applicable.

## Supplementary Materials

The following are available online at https://www.mdpi.com/article/10.3390/biomedicines9091097/s1.
